# First Detection and Molecular Characterization of Peach Latent Mosaic Viroid (PLMVd) in Kazakhstan

**DOI:** 10.3390/pathogens14030243

**Published:** 2025-03-03

**Authors:** Gulshan E. Stanbekova, Leila T. Nadirova, Ruslan V. Kryldakov, Bulat K. Iskakov, Andrey V. Zhigailov

**Affiliations:** 1 M. Aitkhozhin Institute of Molecular Biology and Biochemistry, 86 Dosmukhamedov Str., Almaty 050012, Kazakhstanbulat.iskakov2@gmail.com (B.K.I.); 2 Biology Faculty, Al-Farabi Kazakh National University, 71 Al-Farabi Ave., Almaty 050040, Kazakhstan

**Keywords:** PLMVd, viroid, RNA secondary structure, SNP, stone fruit tree, phylogenetic analysis

## Abstract

Viroids represent obligate plant pathogens composed exclusively of non-protein coding small single-stranded RNAs that cause high economic losses worldwide. A field survey was carried out to assess the incidence of the peach latent mosaic viroid (PLMVd) in southeastern Kazakhstan, the region of the country where fruit trees are mainly grown. Of 246 stone fruit trees, 20 (8.13%) were infected with the PLMVd. The incidence of the PLMVd in the peach (19.23%; 15/78) was significantly higher than that in the apricot (6.76%; 5/74; *p* = 0.0234). Eight of the detected viroids were cloned and used for full-genome sequencing. The nucleotide sequence similarity of the selected isolates found in Kazakhstan was 83.9–100%. A phylogenetic analysis indicated three clusters for the Kazakhstani isolates of the PLMVd. Three groups of Kazakhstani viroids differed in their predicted secondary structure. During the survey, the PLMVd was detected and genetically characterized for the first time in Kazakhstan. The obtained results indicate the need to develop state control measures for the PLMVd, including regular monitoring surveys. We identified several SNPs of the PLMVd that had not been previously described. The results may be useful in optimizing diagnostic approaches for detecting stone fruit viroids and preventing their spread through propagation material.

## 1. Introduction

Viroids are subviral pathogens that infect higher plants, causing significant damage. Their genome consists of single-stranded, highly structured circular RNA that does not code for any protein [1]. Viroids may be spread mechanically through wounding or via vegetative transmission, but there are indications that some viroids can also be transmitted by aphids [2]. In the fruit tree industry, the economic impact of viroids is significant, primarily due to their detrimental effects on fruit quality and the productivity of fruit trees. The mature circular viroids are resistant to RNA silencing-mediated degradation [3]. For this reason, RNA interference-based strategies used to reduce the effects of viroid infections in genetically modified plants can only reduce viroid amplification, but not eliminate these agents [4]. Therefore, the main method of combating viroid infections is the widespread use of molecular diagnostic methods and certified pathogen-free propagation material [5].

Viroids are currently divided into two families: *Avsunviroidae* and *Pospiviroidae*. *Avsunviroidae* members undergo replication in chloroplasts and have a highly branched structure containing hammerhead ribozymes [6], while *Pospiviroidae* members replicate in the nuclei of infected cells and exist as highly base-paired rod-like RNA structures lacking ribozymes [6].

Although several viroid species have been isolated from stone fruit tree specimens, only two species, namely the peach latent mosaic viroid (PLMVd) and the hop stunt viroid (HSVd), are directly involved in a specific disease (consistent with Koch’s postulates) [7]. A member of the *Avsunviroidae* family, the PLMVd is an important economically significant quarantine infectious agent of the peach [8,9]. It reduces the tree yield; stunts the tree growth; shortens the lifespan; causes mosaic and leaf spots, fruit deformation and discoloration, stem pitting, and delayed leaf emergence; and makes the trees more susceptible to other biotic and abiotic stresses [10]. The peach is the primary host for the PLMVd [10].

For the first time in Kazakhstan, our research group identified the PLMVd in peach trees with moderate clinical symptoms [11]. This indicates the need to conduct a monitoring study and molecular characterization of the domestic isolates of this viroid. The territory of Kazakhstan is mainly represented by semi-arid and arid lands. However, the foothill zone of southeastern Kazakhstan (the Tien Shan and Dzungarian Alatau mountain ranges) has favorable conditions for the successful development of irrigated agriculture and the cultivation of fruit trees. It is in this region of Kazakhstan that stone fruit trees are mainly grown [12]. This study aims to determine the incidence of the PLMVd in stone fruit trees (species of the genus *Prunus*, family Rosaceae) in the southeast of Kazakhstan and its molecular genetic characteristics. To date, field surveys of the viroid diseases of fruit crops in Kazakhstan have not been carried out. This is the first time that the molecular characterization (whole-genome sequencing) of Kazakhstani genetic variants of the PLMVd has been conducted.

## 2. Materials and Methods

### 2.1. Plant Material and RNA Isolation

The southeastern Kazakhstan region accounts for more than 90% of the stone fruit crop production [12]. Peaches are hardly grown in the areas of the country located to the north due to the severe cold winters. For this reason, samples were collected in the southeast to monitor the PLMVd.

We surveyed peach, apricot, cherry, plum, and nectarine trees growing in state and private orchards and nurseries located in the Almaty (five districts), Turkistan (four districts), Jetisu (one district), and Zhambyl (two districts) oblasts of Kazakhstan, as well as in the cities Almaty (three districts) and Shymkent (two districts) for the presence of PLMVd (Table 1). The sample collection periods were August/October 2023 and June/September 2024.

**Figure 1:**
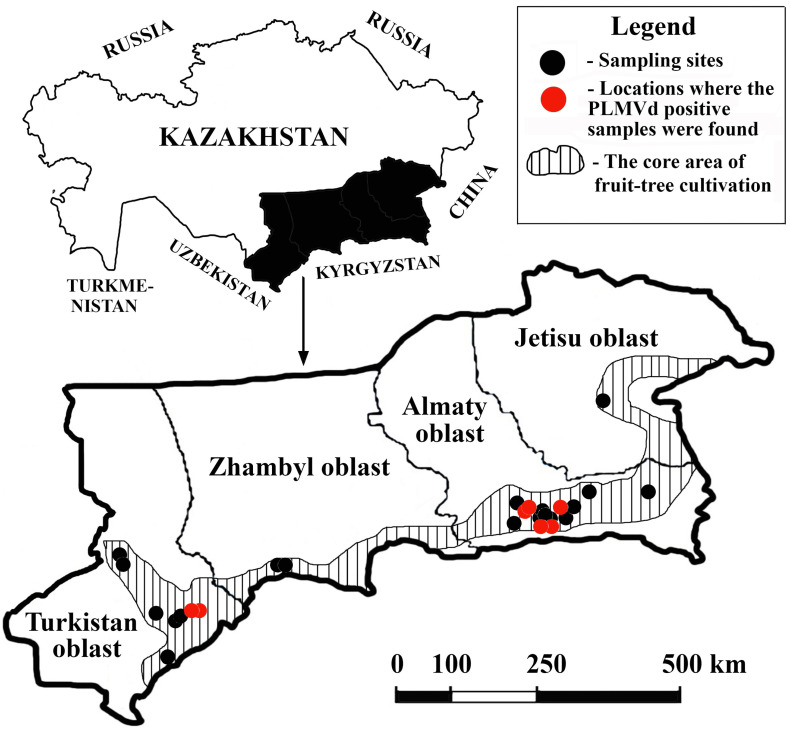
Sampling sites in southeastern Kazakhstan included in this survey.

We used a semi-targeted approach for sampling. Some locations, such as the botanical garden (“Botany garden” locality), arboretum (“Dendarium” locality), nurseries (“Tastybulak”, “Atbulak”, and “Sayram” localities), and peach orchards (“Jemisty” and “Chundzha” localities), were chosen deliberately due to their good representation of stone fruit trees. The remaining 20 localities were randomly selected from 1278 settlements within the core area of fruit tree cultivation in the country (Figure 1) using the RANDBETWEEN function in MS Excel 2016.

In one locality, a maximum of three different sampling sites were used. Preference was given to those collection sites in one locality that contained peaches. Visits were only conducted if the owners of the homesteads or private orchards agreed to participate in the survey. At certain collection sites, samples were collected regardless of the symptoms displayed. At one collection site, no more than five stone fruit trees of one species were selected; we tried to maintain uniform intervals between the trees selected for sampling, but the intervals were not less than five meters (regardless of the type of tree). From each tree, 3–5 leaves were collected from different parts of the crown. The geographic locations of the selected sampling sites are shown in Figure 1.

Cetyltrimethylammonium bromide (CTAB) (Fluka) was used to extract the RNA [13] from 0.3 g of fresh or frozen leaf samples collected from 246 stone fruit trees, including peach (78), apricot (74), cherry (64), plum (27), and nectarine (3) trees. Although the peach is a minor stone fruit crop in Kazakhstan, it is the peach that is the main host of the PLMVd. Therefore, samples were mainly collected from peach trees for analysis.

### 2.2. Reverse Transcription and PCR

First-strand cDNA was synthesized from the RNA using Maxima Reverse Transcriptase (Thermo Fisher Scientific, Waltham, MA, USA) according to the manufacturer’s instructions. Mixtures containing 4.5 μL of sample RNA (300–500 ng) and 0.5 μL (100 μM) of random hexamer primers (Thermo Fisher Scientific, Waltham, MA, USA) were heated at 65 °C for 5 min and then cooled on ice. Then, 6.5 μL of reverse transcription solution containing 2 μL of 5× reverse transcription buffer (Thermo Fisher Scientific, Waltham, MA, USA), 1 μL of 10 mM dNTP mixture, 0.25 μL (40 units) of Maxima Reverse Transcriptase (Thermo Fisher Scientific, Waltham, MA, USA), and 0.25 μL (10 units) of ribonuclease inhibitor (Thermo Fisher Scientific, Waltham, MA, USA) were added to each reaction. The temperature conditions were as follows: 25 °C for 10 min; 50 °C for 30 min; 85 °C for 5 min.

A conventional RT-PCR for the detection of the PLMVd was performed using the primers plmf (5′-GGATTACGACGTCTACCCGG-3′) and plmr (5′-CCAGTTTCTACGGCGGTACCTG-3′), which amplifies the full genome (~338 nt.) of this viroid [14]. These primers were designed based on reference GenBank sequences (GenBank: MN857143, LC333096, KF870129, MF574159, EU708826, KX430172, MH974836, MK212065, PP079191, MW928676, MG788244, OR576774, MZ220887, KU048780, M83545).

The viroid cDNAs were amplified using Pfu DNA polymerase (Thermo Fisher Scientific, Waltham, MA, USA) according to the manufacturer’s instructions. Each 25 μL PCR contained 2.5 μL of the reverse transcription reaction product, 2.5 μL of 10× Standard Pfu Buffer with MgSO_4_ (Thermo Fisher Scientific, Waltham, MA, USA), 0.5 μL of 10 mM dNTPs (Thermo Fisher Scientific, Waltham, MA, USA), 0.6 μL of a 10 μM solution of each primer, 0.5 μL (1.25 U) of Pfu DNA Polymerase (Thermo Fisher Scientific, Waltham, MA, USA), and 17.8 μL of sterile water for the PCR (Biolabmix, Novosibirsk, Russia). The temperature profile of the PCR amplification was as follows: 95 °C for 2 min, 95 °C for 30 s, 58 °C for 30 s, and 72 °C for 1 min; 35 cycles were performed followed by 5 min of incubation at 72 °C. The PCR products were analyzed through electrophoresis in a 1.8% TBE agarose gel, stained with ethidium bromide, and visualized under UV light.

### 2.3. cDNA Cloning and Sequencing

The RT-PCR products containing full genomes of the PLMVd were obtained by using Pfu polymerase, and subsequent adenine nucleotide tailing were ligated into the pBluescript SK II(+) cloning vector that has been cleaved with *EcoR*V and tailed with extra T at 3′-ends (TA-cloning) [15]. The cloned PLMVd cDNAs were sequenced in both directions using M13 universal primers and the BigDye™ Terminator v.3.1 kit (Applied Biosystems), according to the manufacturer’s recommendations, and analyzed using a 24-capillary ABI 3500XL Gene Analyzer (Applied Biosystems, Foster City, CA, USA). If several positive PLMVd samples were identified in one sampling site, only one of them was subjected to cloning and sequencing.

### 2.4. Phylogenetic Analysis

The Basic GenBank Local Alignment Search Tool Program (BLAST) (release 264.0) was used to compare the resulting nucleotide sequences with the sequences deposited in the NCBI GenBank (https://www.ncbi.nlm.nih.gov (accessed on 24 February 2024)) and to calculate the statistical significance of matches. Multiple sequence alignment was performed using the MUSCLE algorithm. The phylogenetic relationships among the analyzed isolates were established using maximum-likelihood algorithms and the Tamura–Nei model [16] implemented in the Molecular Evolutionary Genetics Analysis (MEGA) X software ver. 10.1.8 [17]. The statistical significance was estimated using a bootstrap test with 1000 replications.

### 2.5. Prediction of Secondary Structures of Viroid RNAs

The predicted secondary structures according to the minimal free energy of the PLMVd variants were obtained using the online web tool RNAstructure (available at https://rna.urmc.rochester.edu/RNAstructureWeb/index.html (accessed on 24 February 2024)) [18].

### 2.6. Repositories

The newly generated sequences reported in this work are available in the GenBank database (https://www.ncbi.nlm.nih.gov (accessed on 24 February 2024)) under the access numbers: PP857833–PP857834 and PV034720–PV034725.

## 3. Results

### 3.1. Samples

Leaf samples were collected from 246 stone fruit trees from 44 sites in 26 locales across the core area of fruit tree cultivation as a part of the efforts to monitor the health status of stone fruit trees in southeastern Kazakhstan (Table 1 and Figure 1). Most of the samples collected did not show conspicuous symptoms. However, a number of trees exhibited moderate symptoms of the disease, namely cracks in the tree bark (12), a delay in foliation (11), upward leaf curling (8), and fruit deformation (3). In the next growing season, nine trees, including five peach, two apricot, and two cherry, did not produce leaves and died.

### 3.2. Identification of the PLMVd from Stone Fruit Trees

Using PLMVd-specific primers, we examined all 246 RNA samples using RT-PCR. The RT-PCRs amplified specific products of approximately 340 bp in size for 20 samples (8.13%; 95% CI = 5.04–12.28%) of stone fruit trees. The incidence of PLMVd was estimated to be 19.23% (15/78; 95% CI = 11.18–29.73%) in peach trees and 6.76% in apricot trees (5/74; 95% CI = 2.23–15.07%). Therefore, the incidence of PLMVd in the peach is significantly higher than that in the apricot (χ^2^ = 5.1370; *p* = 0.0234). No positive PLMVd samples were detected in cherry and plum trees. Ten out of twenty PLMVd-positive trees (50%, 95% CI = 27.2–72.8%) had at least one of the following symptoms: bark cracking, death in a year, delay in foliation, and fruit deformation. The other ten PLMVd-infected trees did not show any symptoms.

### 3.3. Phylogenetic Analysis of the Identified PLMVd Variants

**Figure 2:**
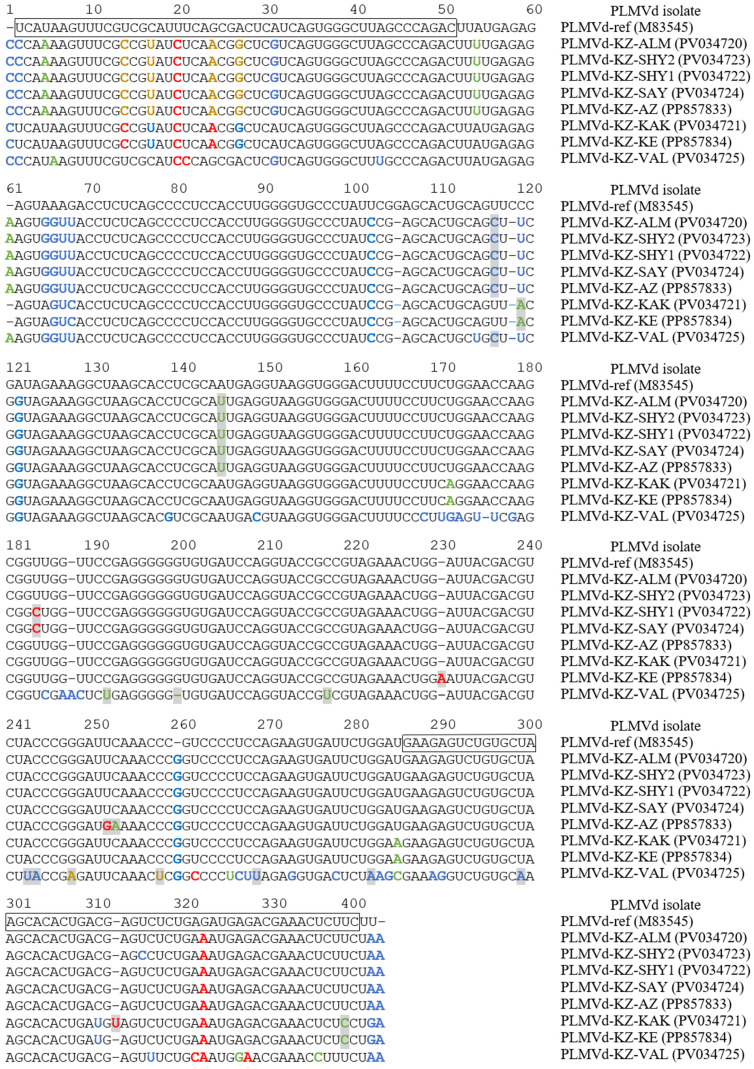
The alignment of cloned whole-genome sequences of the PLMVd from southeastern Kazakhstan. The PLMVd reference sequence (GenBank: M83545) [19] is shown for comparative purposes. Nucleotides involved in the formation of plus and minus hammerhead structures are boxed [20]. Nucleotide variations identified only in Kazakhstani isolates are indicated with gray boxes. Nucleotide variations that disrupt the basic structures P1–P11 of the PLMVd are highlighted in red. Nucleotide variations that restore or enhance the basic structures of the PLMVd are highlighted in blue. Nucleotide variations that do not affect the formation of secondary structures of the PLMVd are highlighted in green. Nucleotide variations that disrupt the basic structures of the PLMVd but contribute to the formation of other secondary structures are highlighted in light brown.

To verify the PCR results, we cloned eight of the generated amplicons into the pBluescript KSII vector using the TA-cloning technique, followed by the dideoxy Sanger sequencing of inserts using M13 and T7 primers to obtain the complete PLMVd genomes (Table 2). Nucleotide comparisons in the BLAST revealed all the cloned amplicons compared to the PLMVd. The multiple sequence alignment of the full-lengths of the PLMVd clones is presented in Figure 2.

The PLMVd variants were very diverse, showing 82.2–95.5% identity to the reference genome. The alignment analysis showed several mutations that had never been previously described (Figure 2).

**Figure 3:**
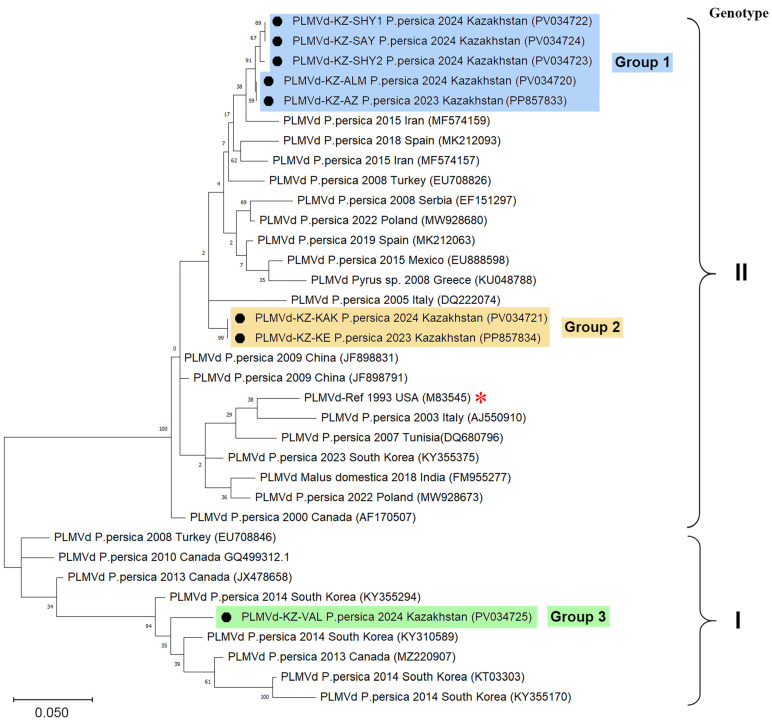
Phylogenetic analysis based on the full-genome sequences of PLMVd isolates. The neighbor-joining phylogenetic tree is constructed in MEGA-X from alignments of eight complete PLMVd sequences generated in this study and 27 database sequences. The tree is drawn to scale, with the branch lengths representing the numbers of substitutions per site. The percentage of trees in which the associated taxa clustered is shown next to the branches. The GenBank accession numbers are shown in parentheses. The Kazakhstani PLMVd isolates determined in this study (marked with a black circle) are in blue, orange, or green rectangles, depending on their grouping. The red asterisk is the reference genome (GenBank: M83545) [19].

We constructed a phylogenetic tree to reveal the phylogenetic relationships of the identified PLMVd with other known isolates (Figure 3). A phylogenetic analysis of the full Kazakhstani PLMVd genomes led to their clustering into three major groups with a low mean nucleotide intragroup divergence reaching 1%. The isolates from PLMVd Group 1 showed moderate similarity (95.8% on average) to those from Group 2. At the same time, the isolate PLMVd-KZ-VAL (PV034725), forming Group 3, showed a relatively low sequence similarity to isolates from PLMVd Group 2 and Group 3, at 86.6% and 83.9% on average, respectively. This isolate of the PLMVd is related to genotype I PLMVd (according to Ambrós et al. [21]). It is characterized by a genome 337 nt in length, which distinguishes it from other Kazakhstani isolates, whose length was 338 nt. Viroids of Group 1 (GenBank: PP857833, PV034720, PV034722–PV034724) and Group 2 (GenBank: PP857834, PV034721) were classified as genotype II PLMVd (according to Ambrós et al. [21]), but clustered into distinct clades (Figure 3).

Representatives of Group 1 PLMVd were identified in four districts of the Almaty and Turkistan oblasts, while representatives of the other two groups were identified only within the city of Almaty. Isolates from Group 1 were genetically closest to the isolates from Iran (GenBank: MG788245, MF574157, MF574159) and Poland (GenBank: MW928681). Representatives of Group 2 were evolutionarily most related to the isolates from Spain (GenBank: AJ005316, AJ005317, MK212068). The only representative of Group 3 (GenBank: PV034725) was evolutionarily related to isolates from South Korea (GenBank: KY355291, KY355294, KY355296).

None of the PLMVd-positive samples showed leaf mosaic symptoms in the peach; however, 6 of the 20 variants were associated with a delay in foliation and cracked bark. Three peach trees, in the leaves of which the PLMVd was identified, died in a year (Table 2). Due to the small number of identified genetic variants, there was no opportunity to establish a correlation between the nucleotide sequence of individual PLMVd isolates and symptoms.

### 3.4. RNA Secondary Structure

**Figure 4:**
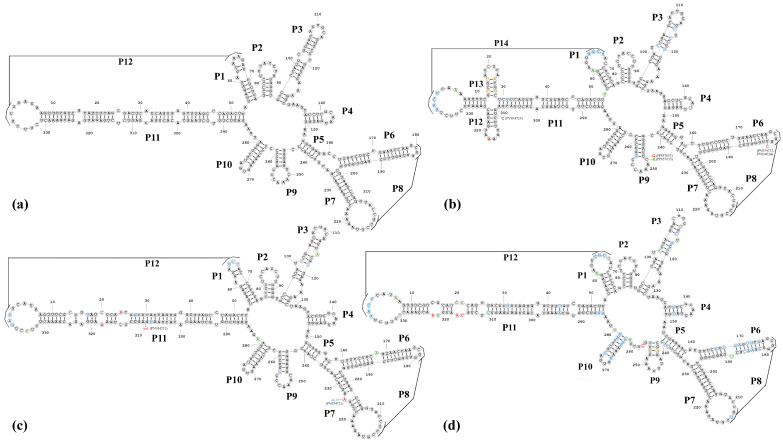
Nucleotide sequences of the Kazakhstani PLMVd variants and the reference isolate of PLMVd folded in the secondary structure of the lowest free energy predicted by the RNA-structure prediction tool [18]. (**a**) The PLMVd reference structure (GenBank: M83545) [19]; (**b**) Group 1 Kazakhstani PLMVd isolates (GenBank: PP857833, PV034720, PV034722-PV034724); (**c**) Group 2 Kazakhstani PLMVd isolates (GenBank: PP857834, PV034721); (**d**) Group 3 Kazakhstani PLMVd isolates (GenBank: PV034725). Nucleotide variations that disrupt the basic structures of the PLMVd are highlighted in red. Nucleotide variations that restore or enhance the basic structures of the PLMVd are highlighted in blue. Nucleotide variations that do not affect the formation of secondary structures of the PLMVd are highlighted in green. Nucleotide variations that disrupt the basic structures of the PLMVd but contribute to the formation of other secondary structures are highlighted in light brown.

Various variants of viroids can only survive if the changes in their nucleotide sequences do not disrupt the formation of functionally significant secondary and tertiary structures [22,23]. We analyzed the relationship between nucleotide variations and the predicted RNA secondary structures. The most stable secondary structures (with the lowest free energies) were predicted for all the sequenced PLMVd variants using the RNA-structure online server of Mathews Lab Home (available at https://rna.urmc.rochester.edu). The PLMVd reference sequence (GenBank: M83545) [19] was taken for comparative purposes. For a better description of secondary structure, the helices (P1–P14) of the PLMVd secondary structures were numbered as per Perreault et al. [24]. The results of the analysis are presented in Figure 4.

Analysis of the various mutations found within the PLMVd sequences showed that viroids that clustered into the same clades during the phylogenetic analysis had similar secondary structures. The predicted secondary structure of Group 1 viroids included 14 main helices (P1–P14) and most closely resembled the PLMVd structure described by Perreault et al. (2024) (Figure 4b). The secondary structure of two isolates from the Group 2 (GenBank: PP857834, PV034721) viroids (Figure 4c) included 12 helices (P1–P12) and was similar to that of the reference PLMVd isolate (GenBank: M83545) (Figure 4a). We found several nucleotide variations (Figure 4c), but the structure of the viroids was not affected by these single nucleotide polymorphisms (SNPs). The isolate representing Group 3 PLMVd (GenBank: PV034725) is of significant interest because, despite the significant difference in its nucleotide sequence from the reference genome of PLMVd (82.2% identity), most of the SNPs did not disrupt, restore, or form similar secondary P1–P12 helices (Figure 4d). It should be noted that, in helix P5 of this viroid, a shift occurs during the formation of hydrogen bonds, and instead of helix P9, an analogous but completely different loop structure is formed (Figure 4d).

## 4. Discussion

This study showed that the PLMVd occurred in southeastern Kazakhstan in stone fruit trees of at least two species (peach and apricot). The genetic characteristics of the full-genome sequences of the selected PLMVd isolates found in different regions were analyzed for the first time in Kazakhstan. These isolates significantly differed in length, nucleotide sequences (they were clustered into three main groups), and predicted secondary structures. The phylogenetic analysis of the Kazakh isolates of PLMVd revealed several evolutionarily related isolates of this viroid from various countries and regions of the world, which makes it unlikely for the viroids to have been introduced into the country from a single source.

In the Jetisu, Almaty, and Zhambyl oblasts, peaches and apricots are mainly grown in private households for personal consumption, while in the Turkistan oblast of Kazakhstan, peaches are grown on a large scale and mainly for sale. The fact that we detected the presence of the PLMVd in the Turkistan oblast indicates significant potential problems that may arise for farmers engaged in the cultivation of these stone fruit crops.

Representatives of all three groups of PLMVds were identified within the Almaty city in locations that are relatively close to each other. This is not surprising, as the city of Almaty and the adjacent areas of the Almaty oblast are the leaders in the import of planting material for fruit trees from abroad. Several large nurseries of fruit trees are located here.

The globalization of trade, the intensification of transportation, and climate change contribute to the spread of dangerous infectious agents of plants to non-endemic regions. Molecular-genetic approaches based on RNA interference that have proven effective in protecting plants from viruses [25] turn out to be of little use in protecting against viroids [4,26]. Currently, the only practical way to control viroid infections is through regular monitoring surveys to identify infection hotspots and their localization and the thorough testing of planting material for the presence of viroids [1].

Although the PLMVd is considered a quarantine infectious agent in the territory of the Eurasian Economic Union (EAEU) [27], there is currently no control over imported planting material and the state monitoring of the PLMVd in the territory of Kazakhstan. This significantly increases the risks of new dangerous strains of this hazardous infectious agent entering the country. To the best of our knowledge, the PLMVd had not been previously detected in the territory of the EAEU. The results we obtained indicate the need for regular monitoring surveys. This particularly applies to Uzbekistan, which borders the Turkistan oblast, and Kyrgyzstan, which borders the Almaty oblast, where circulation of the PLMVd has been shown. The conducted genetic characterization of the PLMVd isolates identified in the territory of Kazakhstan may improve diagnostic approaches for identifying this viroid.

According to the Common Quarantine Phytosanitary Requirements of the EAEU (Decision of the EEC Council of 30 November 2016 No. 157), the planting material for peaches must come from zones free from the PLMVd. This should lead to an increase in phytosanitary control over fruit trees in the Almaty and Turkistan oblasts of Kazakhstan. In districts where the presence of the PLMVd has been detected, a quarantine would be imposed on the export of planting material for stone fruit trees. Visual inspection of fruit trees is insufficient to prevent the spread of the PLMVd, because most of the infected stone fruit trees did not show any conspicuous symptoms [9]. We have shown that the method we used to detect the PLMVd, based on conventional RT-PCR, is quite suitable for identifying Kazakhstani isolates of this viroid in plant material. One of the challenges in controlling viroid diseases is the insufficient awareness of farmers regarding the etiology, symptoms, and routes of transmission of these infections. We will try to release recommendations for farmers that will provide information about the threat that the PLMVd poses to fruit growing, caused by its symptoms, as well as ways to control PLMVd infection.

Using pathogen-free propagation material will be a basic approach through which the fruit crop industry can prevent the spread of the PLMVd. We will do our best to ensure that information about the circulation of the PLMVd reaches the owners of fruit tree nurseries in the southeastern region. We hope that the results we have obtained and further work on improving the effectiveness of PLMVd infection control will help to reduce PLMVd transmission into non-endemic territories.

This work has several limitations. We were only able to analyze a rather small number of samples, which did not allow us to determine whether the features of the nucleotide sequences and secondary structures of the viroids correlates with the pathogenic properties of the PLMVd. The mechanical inoculation of the control peach trees with PLMVd isolates was not performed. For this reason, it was impossible to conduct transmission studies to determine the possible routes of PLMVd spread and to establish a correlation between symptomatological/phytopathological variation in various groups of viroids. The semi-targeted approach used for sampling increased the likelihood of detecting the PLMVd, but led to significant overestimation of the calculated prevalence of PLMVd in the studied area.

## 5. Conclusions

We analyzed the incidence of PLMVd in Kazakhstan for the first time and determined the genetic characteristics of Kazakhstani PLMVd isolates. Studying the genetic variability in the nucleotide sequences of viral and viroid pathogens is important for epidemiology and control purposes. We identified several SNPs of the PLMVd that had not been previously described. The results may be useful for improving the effectiveness of diagnostic approaches for detecting stone fruit viroids and preventing their spread through propagating material.

## Figures and Tables

**Table 1:** Characteristics of sampling locations.

No.	Oblast	District	Locality	Coordinates	Source				
					Peach	Apricot	Nectarine	Plum	Cherry
1	Almaty oblast	Karasay	Almalybak	43.219653, 76.680165	2	4		2	7
2		Karasay	Zhalpaksay	43.237491, 76.682113	1	2		1	2
3		Karasay	Korgauldy	43.168614, 76.760671	3				
4		Karasay	Zhandosovo	43.164162, 76.555497	5	5			5
5		Karasay	Shamalgan	43.366580, 76.637612		5		3	5
6		Enbekshikazakh	Esik	43.357364, 77.458232		5		1	3
7		Enbekshikazakh	Saymasay	43.446146, 77.328007	2				
8		Enbekshikazakh	Karaturyk	43.556985, 77.994449	4	5	3	3	7
9		Talgar	Baibulak	43.282523, 77.191661	2				
10		Talgar	Besagash	43.289931, 77.060820	1	5			5
11		Ile	Boralday	43.370097, 76.872323	2				
12		Uygur	Chundzha	43.524600, 79.474040	10				
13	Almaty ^1^	Nauryzbai	Karagaily	43.161956, 76.847858	5	3			3
14		Nauryzbai	Tastybulak	43.172998, 76.81184	4	2			
15		Medeu	Ak-Kaiyn	43.173749, 76.967011	2	5			
16		Bostandyq	Botany garden	43.223860, 76.914160	5				
17	Jetisu oblast	Karatal	Ushtobe	45.246485, 77.987515		4		2	3
18	Zhambyl oblast	Taraz	Taraz	42.864126, 71.410216		4		3	2
19		Zhambyl	Zhambyl	42.993133, 71.206504		3		2	1
20	Shymkent ^1^	Karatau	Sayram	42.297949, 69.716074	2	2			
21		Shymkent	Dendarium	42.367953, 69.612962	5	5		2	2
22	Turkistan oblast	Saryagash	Jemisty	41.491492, 69.316311	10	7		8	8
23		Turkistan	Birlik	43.333654, 68.247169	3				
24		Turkistan	Turkistan	43.289631, 68.345815		2			3
25		Ordabasy	Badam	42.393802, 69.251972	3				
26		Kazygurt	Akzhar	42.234787, 69.492169	2	6			5
27		Kazygurt	Atbulak	42.345041, 69.492893	5				3
Total:					78	74	3	27	64

^1^ Almaty and Shymkent are cities of regional significance.

**Table 2:** List of the Kazakhstani PLMVd isolates with some characteristics.

Isolate	Accession Number	Host	Locality	PLMVd Sequence Analysis			Symptoms ^1^
				Group	Length	New SNPs	
PLMVd-KZ-ALM	PV034720	peach	Almalybak	1	338 nt	U115C, A143U	CB, DF
PLMVd-KZ-AZ	PP857833	peach	Zhalpaksay	1	338 nt	U115C, A143U, U248G, C249A	CB, D, DF
PLMVd-KZ-SAY	PV034724	peach	Saymasay	1	338 nt	U115C, A143U, U182C	-
PLMVd-KZ-SHY1	PV034722	peach	Dendarium	1	338 nt	U115C, A143U, U182C	-
PLMVd-KZ-SHY2	PV034723	peach	Sayram	1	338 nt	U115C, A143U	D, FD
PLMVd-KZ-KAK	PV034721	peach	Karagaily	2	338 nt	U116A, -309A, U334C	-
PLMVd-KZ-KE	PP857834	peach	Ak-Kaiyn	2	338 nt	U116A, -226A, U334C	DF
PLMVd-KZ-VAL	PV034725	peach	Korgauldy	3	337 nt	U115C, C189U, G196-, C213U, A238U, C239A, G243A, C253U, G277A, U294A	CB, D, DF

^1^ CB = cracks in the tree bark; D = death in a year; DF = delay in foliation; FD = fruit deformation.

## Data Availability

The nucleotide sequences reported in this paper are deposited into the NCBI GenBank database (www.ncbi.nlm.nih.gov (accessed on 24 February 2024)) under the accession numbers listed in the text.

## Abbreviations

The following abbreviations are used in this manuscript:

BLAST	Basic GenBank Local Alignment Search Tool
CI	Confidence interval
EAEU	The Eurasian Economic Union
HSVd	Hop stunt viroid
MEGA	Molecular Evolutionary Genetics Analysis
PLMVd	Peach latent mosaic viroid
SNP	Single nucleotide polymorphism
